# Integrating trauma-informed principles into collaborative care for depression in primary care

**DOI:** 10.3389/frhs.2026.1961806

**Published:** 2026-09-30

**Authors:** Veronica Vitriol

**Affiliations:** Department of Medicine, Faculty of Medicine, Universidad de Talca, Talca, Chile

**Keywords:** childhood maltreatment, collaborative care, depression, primary care, trauma-informed care

## Abstract

Depression remains one of the leading causes of disability worldwide despite the availability of effective pharmacological and psychological treatments. Collaborative care has consistently improved depression outcomes through coordinated, multidisciplinary, and measurement-based approaches, particularly in primary care. However, substantial heterogeneity in treatment response persists, especially among adults with histories of childhood maltreatment, who frequently present with earlier illness onset, greater symptom severity, increased psychiatric comorbidity, poorer treatment response, and a more chronic clinical course. Although collaborative care is effective, assessment of childhood maltreatment and trauma-informed developmental formulation are not consistently integrated into routine depression management within these models. In this Perspective, we propose Trauma-Informed Collaborative Care (TICC) as a conceptual framework for integrating trauma-informed and developmental principles into collaborative care for depression. Rather than replacing existing evidence-based interventions, TICC seeks to strengthen collaborative care by incorporating trauma-informed assessment, developmental case formulation, trusting therapeutic relationships, interdisciplinary supervision, recognition of patients’ strengths and resilience, and organizational practices that promote safety, trustworthiness, collaboration, and empowerment. We review complementary bodies of evidence that provide the rationale for this approach, summarize findings from a recent cluster randomized controlled trial conducted in Chile, and discuss key implementation challenges and future research priorities. Taken together, these distinct bodies of evidence provide a rationale for integrating trauma-informed principles into collaborative care, particularly for patients with histories of childhood maltreatment and greater clinical complexity, while the incremental clinical benefit of this integration within the proposed TICC framework remains to be established.

## Introduction

Depression is one of the leading causes of disability worldwide, affecting approximately 6%–10% of the population annually and nearly one in five individuals over their lifetime (1). Its global burden increased substantially during the COVID-19 pandemic, and in 2023, major depressive disorder ranked as the 15th leading cause of disability-adjusted life years (DALYs) globally among the 304 diseases and injuries included at level 4 of the Global Burden of Disease hierarchy (2). Current projections indicate that the global burden of depression will continue to increase over the coming decades, posing a major challenge for mental health systems worldwide (3, 4).

Despite the availability of several evidence-based pharmacological and psychotherapeutic treatments, a substantial proportion of individuals with depression continue to experience persistent symptoms, recurrent episodes, chronicity, or limited treatment response (5). Among models of depression care, collaborative care has consistently demonstrated superior outcomes compared with usual care by improving depressive symptoms, treatment adherence, and patient satisfaction through coordinated, multidisciplinary, and measurement-based care, particularly in primary care (5–8). Nevertheless, considerable heterogeneity in treatment response persists, especially among individuals with histories of childhood adversity or maltreatment, who frequently present with greater clinical complexity, psychiatric comorbidity, suicidality, and poorer treatment outcomes (9). These findings suggest that optimizing depression care for this population may benefit from approaches that incorporate trauma-informed principles (10).

Although collaborative care provides an effective framework for delivering evidence-based depression treatment, conventional models do not systematically incorporate current evidence regarding childhood maltreatment as a clinically relevant factor associated with differences in clinical presentation, illness course, and treatment response among individuals with depression (9, 11). Integrating this evidence into collaborative care may therefore contribute to a more comprehensive understanding of patients' clinical needs and inform assessment, case formulation, treatment planning, and follow-up.

In this Perspective, we propose Trauma-Informed Collaborative Care (TICC) as a conceptual framework for strengthening collaborative care through the explicit integration of trauma-informed and developmental principles. Rather than replacing established evidence-based approaches, TICC is intended to complement collaborative care by incorporating trauma-informed assessment, developmental case formulation, therapeutic relationships that emphasize emotional safety and trust, recognition of patients' strengths and resilience, interdisciplinary supervision, and care strategies responsive to the needs of individuals with depression and histories of childhood maltreatment. We examine the rationale for this integration, describe the proposed core components of TICC, consider the emerging empirical and implementation evidence, and identify priorities for future research.

This Perspective draws on a narrative, non-systematic synthesis of literature relevant to collaborative care for depression, childhood maltreatment and its clinical consequences, trauma-informed care (TIC), and their integration in primary care. Priority was given to systematic reviews, meta-analyses, and major clinical and epidemiological studies. The synthesis informs the conceptual argument and is not intended as a comprehensive or systematic review.

### Childhood maltreatment: a clinically relevant dimension of depression

Childhood adversity encompasses a broad range of adverse experiences occurring before the age of 18 years. Among these, childhood maltreatment—including physical, emotional, and sexual abuse, as well as physical and emotional neglect—has been particularly strongly associated with adult depression, increasing the risk of developing the disorder by approximately 2.5- to 4-fold (9, 12, 13). Nearly half of adults with depression report a history of childhood maltreatment, with emotional abuse and neglect consistently showing the strongest associations with depressive disorders (9, 13). Depression associated with childhood maltreatment may be characterized by a distinct clinical profile, including earlier onset, greater symptom severity, higher psychiatric comorbidity, a more chronic and recurrent course, and poorer treatment response (9, 13–17). Some of these clinical characteristics are also frequently observed in difficult-to-treat depression (DTD); however, this overlap should not be interpreted as indicating that the two constructs represent the same clinical phenotype. DTD is a broader construct defined by persistent burden despite usual treatment efforts and should also be distinguished from treatment-resistant depression (TRD), which is more narrowly defined in relation to inadequate response to adequately delivered antidepressant treatments (18–20).

Childhood maltreatment, particularly during sensitive developmental periods, has been associated with dysregulation of the hypothalamic-pituitary-adrenal axis, persistent low-grade inflammation, and structural and functional differences in fronto-limbic circuits involved in emotion regulation (21, 22). These differences may partly reflect cumulative developmental stress and contribute to enduring vulnerability (23). Psychologically, childhood maltreatment has been associated with difficulties in attachment, mentalization, emotion regulation, and epistemic trust, potentially contributing to interpersonal difficulties, negative self-representations, maladaptive coping, and recurrent depression (24–26). Together, these findings provide a developmental framework for understanding the clinical profile of depression associated with childhood maltreatment (27).

Despite accumulating evidence, childhood maltreatment remains insufficiently explored in routine mental health assessment and clinical formulation (28, 29). The limited agreement between prospective and retrospective assessments of childhood maltreatment represents an important methodological limitation, indicating that these approaches should not be treated as interchangeable measures of exposure (30). This limits the extent to which findings based on retrospective self-report can be generalized to prospectively documented maltreatment, but such reports may provide clinically relevant information about patients’ recollections and subjective appraisals, complementing symptom-based assessment and informing present-focused case formulation and treatment planning.

### Limitations of conventional depression treatment and opportunities to expand collaborative care

Despite the availability of evidence-based pharmacological and psychological treatments for depression, clinical outcomes remain suboptimal for a substantial proportion of patients (5). Response rates are approximately 50% with either antidepressant medication or psychotherapy alone and increase to around 65% with combined treatment (5). However, response does not necessarily imply remission: a meta-analysis of psychotherapy trials for adult depression reported remission rates ranging from 26% to 34% at approximately two months (31). Greater baseline symptom severity, anxiety comorbidity, and suicidal risk have been associated with treatment resistance (32), while these clinical features are also frequently observed among individuals with histories of childhood maltreatment (9, 13).

Collaborative care is a multicomponent health system intervention that enhances the delivery of depression care through coordinated management involving primary care physicians, case managers, and mental health specialists. Guided by measurement-based care and stepped-care principles, it combines evidence-based pharmacological and psychological treatments with proactive follow-up, regular psychiatric supervision, and systematic outcome monitoring. Multiple randomized controlled trials and meta-analyses have consistently demonstrated that collaborative care improves depressive symptoms, treatment adherence, patient satisfaction, and quality of care compared with usual care, particularly in primary care settings (6–8).

Although collaborative care has substantially improved the organization and delivery of depression care, the growing evidence on the clinical relevance of childhood maltreatment for the presentation, course, and treatment response of depression supports more explicit attention to these experiences in clinical assessment, formulation, and management (11–17). In addition, many elements of TIC—including person-centered care, shared decision-making, therapeutic alliance, continuity, and collaborative relationships—are already consistent with high-quality collaborative care (33). Whether patients with histories of childhood maltreatment derive additional benefit from the systematic integration of trauma-informed principles into collaborative care remains to be established. TICC therefore seeks to build on established collaborative care components by integrating a more explicit trauma-informed and developmental perspective.

One area in which this integration may be relevant is clinical assessment and formulation. Routine assessment appropriately emphasizes current depressive symptoms and measurement-based monitoring, whereas childhood maltreatment and its potential relevance to the patient's current clinical presentation are not routinely explored (28, 29). When clinically relevant, incorporating this developmental information may complement symptom-based assessment and contribute to a broader understanding of factors associated with treatment engagement, interpersonal functioning, and clinical course (34).

A further area concerns workforce training and organizational practice. Care managers and primary care professionals may receive limited formal training in recognizing the potential long-term psychological and interpersonal correlates of childhood maltreatment, trauma-informed communication, and strategies to minimize the risk of retraumatization (34, 35). TICC proposes making these competencies more explicit within collaborative care, together with organizational attention to trauma-informed principles such as safety, trustworthiness, collaboration, and empowerment (10).

### Integrating trauma-informed principles into collaborative care

Building on the rationale outlined above, TICC is conceptualized as an extension of collaborative care in which trauma-informed and developmental principles are explicitly integrated across educational, clinical, supervisory, and organizational domains, while preserving the established core components of collaborative care (Figure 1). The proposed components of this integration are summarized in Table 1 and described below.

**Figure 1:**
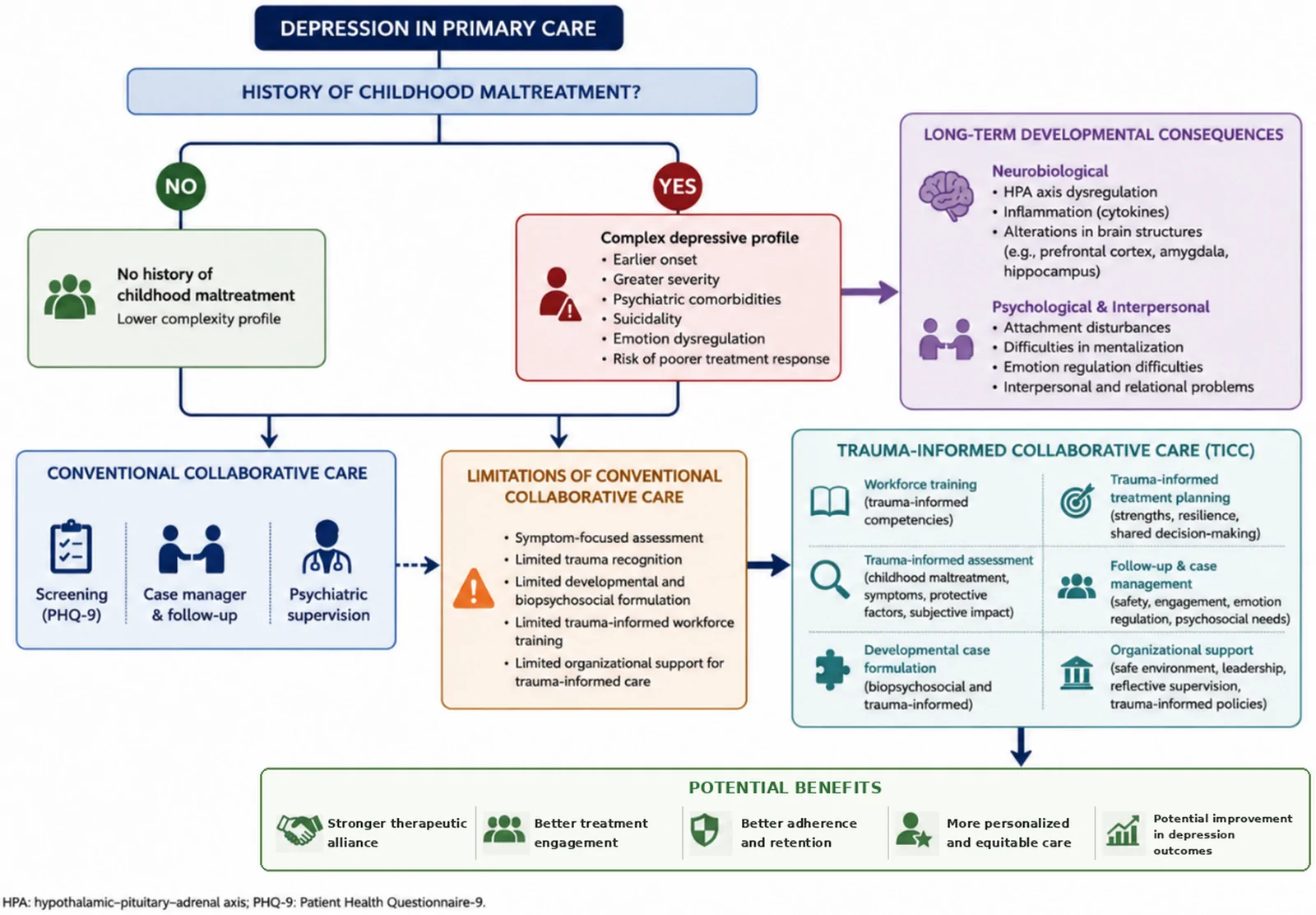
Conceptual framework of Trauma-Informed Collaborative Care (TICC) for depression in primary care. The figure summarizes the clinical complexity and potential long-term developmental consequences associated with childhood maltreatment, limitations of conventional collaborative care in addressing these factors, and the proposed clinical and organizational components of TICC. The benefits presented in the figure are proposed or hypothesized rather than established. The incremental clinical benefit of TICC over conventional collaborative care has not yet been demonstrated, and the available multicomponent trial does not allow the specific contribution of the trauma-informed components to be determined. HPA, hypothalamic-pituitary-adrenal axis; PHQ-9, Patient Health Questionnaire-9.

**Table 1:** Comparison of conventional collaborative care and trauma-informed collaborative care (TICC).

Components	Conventional collaborative care	Proposed Trauma-Informed Collaborative Care (TICC) framework
Workforce training	Training in depression management, measurement-based care, and collaborative care principles	Collaborative care competencies complemented by trauma-informed communication, recognition of trauma-related presentations, strategies to minimize the risk of retraumatization, and reflective practice
Clinical assessment and case formulation	Systematic assessment and monitoring of depressive symptoms, severity, comorbidity, and treatment response	Routine measurement-based assessment complemented, when clinically relevant, by assessment of childhood maltreatment, trauma-related symptoms, protective experiences, current stressors, and subjective appraisal of adversity, informing a present-focused developmental biopsychosocial formulation
Treatment planning and therapeutic relationship	Evidence-based pharmacological and psychological treatment guided by shared decision-making	Evidence-based treatment and shared decision-making informed by the developmental formulation, with explicit attention to emotional safety, trustworthiness, engagement, strengths, resilience, and minimizing the risk of retraumatization
Follow-up and case management	Care coordination, proactive follow-up, symptom monitoring, and support for treatment adherence	Standard follow-up and case-management functions complemented, when clinically relevant, by attention to emotional safety, engagement, emotion regulation, psychosocial stressors and needs, and strengths-based support
Psychiatric supervision	Review of diagnosis, treatment plan, medication, treatment response, and need for stepped care	Standard clinical review complemented by developmental formulation, attention to interpersonal dynamics and treatment engagement, risk of retraumatization, strengths and protective factors, and reflective practice
Organizational approach	Defined team roles, coordinated workflows, measurement systems, and pathways for stepped care	Collaborative care infrastructure complemented by trauma-informed leadership, emotionally safe environments, staff well-being, reflective supervision, and policies emphasizing safety, trustworthiness, collaboration, empowerment, and cultural responsiveness

#### Workforce education and training

Within the proposed TICC framework, workforce education and training would include not only knowledge of childhood maltreatment but also competencies in trauma-informed communication, recognition of trauma-related presentations, collaborative decision-making, strategies to minimize the risk of retraumatization, and reflective practice. Ongoing education and reflective supervision may help sustain these competencies and support consistent implementation across providers (10, 36).

#### Screening and assessment

Collaborative care emphasizes systematic assessment of depressive symptoms and measurement-based monitoring using standardized instruments such as the PHQ-9 (8). TICC could complement this approach by incorporating developmentally informed assessment of childhood maltreatment when clinically relevant, including consideration of current psychosocial stressors, trauma-related symptoms, protective experiences, and the patient's subjective appraisal of adversity (37, 38). Such assessment should inform clinical formulation and individualized care rather than constitute an end in itself. Importantly, TICC does not necessarily imply universal ACE screening, as evidence that systematic screening improves clinical outcomes in adults remains limited, and developmentally informed assessment should complement rather than replace routine measurement-based monitoring (39, 40).

#### Treatment planning, follow-up, and case management

Treatment planning could complement symptom- and treatment-focused approaches with a trauma-informed, developmentally informed, and present-focused biopsychosocial formulation that considers childhood adversity, attachment and interpersonal patterns, emotion regulation, strengths, resilience, and patient preferences within shared decision-making. Such a formulation may help relate relevant developmental experiences to current symptoms, treatment engagement, coping strategies, and interpersonal functioning, while maintaining a clear focus on the patient's current needs, priorities, and treatment goals. During follow-up, routine symptom and treatment monitoring could be complemented, when clinically relevant, by attention to emotional safety, treatment engagement, emotion regulation, adherence, and changes in psychosocial stressors, allowing care plans to be adapted to patients' evolving clinical and psychosocial needs (35).

#### Psychiatric supervision

Psychiatric supervision could make trauma-informed and developmental formulation more explicit by considering the potential clinical relevance of childhood maltreatment to the patient's current presentation, interpersonal dynamics, and treatment engagement, while also attending to the risk of retraumatization and identifying strengths, protective factors, and opportunities to support resilience and recovery. Reflective supervision may further support trauma-informed learning, clinical reflection, and multidisciplinary practice (41).

#### Organizational level

At the organizational level, the proposed TICC framework would emphasize emotionally safe care environments, staff well-being, trauma-informed leadership, and reflective supervision, while embedding relevant trauma-informed principles within organizational culture, policies, and clinical workflows (35). Implementing these components may increase organizational complexity and require additional resources, training, supervision, and attention to implementation fidelity (35, 36). Their feasibility is likely to vary across health systems, particularly in resource-constrained settings, and the clinical benefit of systematically integrating these components into routine care has not yet been established (42–45).

### Emerging evidence and implementation experiences

TIC has been implemented across mental health, primary care, and community settings (42, 45). Although evidence remains heterogeneous, systematic reviews and meta-analyses suggest possible improvements in patient engagement, therapeutic relationships, perceived safety, service satisfaction, coercive practices, and staff burnout (42–45). However, few studies have used rigorous experimental designs, and most implementation research comes from specialized mental health or social service settings in high-income countries, limiting generalizability to primary care and low- and middle-income countries (42–45).

Taken together, the evidence supporting collaborative care, the evidence linking childhood maltreatment with the clinical complexity of depression and treatment outcomes, and the broader evidence for TIC provide complementary but distinct foundations for integrating trauma-informed principles into collaborative care (8, 9, 13, 42–45). Direct evidence supporting this integration remains limited; however, preliminary evidence from primary care suggests that trauma-informed principles can be incorporated within a collaborative care intervention and implemented in routine clinical practice (43).

Chile offers a relevant context for examining this integration because depression is a national priority within primary health care (46). Over the past two decades, Chilean studies have documented a high prevalence of childhood maltreatment among adults receiving treatment for depression in primary care, affecting approximately 60%–80% of patients (47). Although higher than the approximately 50% reported internationally, these estimates derive from selected clinical samples and may also reflect differences in definitions and measurement approaches (9, 13). Nevertheless, they are broadly consistent with other Chilean evidence documenting a high burden of childhood adversity and maltreatment, including studies using the ACE-IQ and national surveys of child and adolescent victimization (48–50).

Evidence from Chilean clinical samples of adults with depression has also shown that, compared with individuals without such histories, those reporting childhood maltreatment present a more severe and clinically complex profile, characterized by earlier onset of depression, greater symptom severity, higher rates of psychiatric comorbidity and suicidality, and poorer treatment outcomes (51–54). Together, these findings support the rationale for depression care models that recognize and address the developmental and clinical consequences associated with childhood maltreatment (55).

In this context, a cluster randomized controlled trial conducted in the Maule Region of Chile evaluated a multicomponent collaborative care intervention for adults with depression in primary care. The intervention combined trauma-informed workforce training, multidimensional assessment of childhood maltreatment, developmental case formulation, proactive follow-up, and regular interdisciplinary supervision, with an emphasis on safe and trusting therapeutic relationships, patients' strengths and resilience, and collaborative, person-centered care alongside evidence-based pharmacological and psychological treatment (56).

At six months, participants receiving the intervention showed greater improvement in depressive symptoms, measured with the Patient Health Questionnaire-9 (PHQ-9), than those receiving usual care (adjusted mean difference [AMD] = −3.09, 95% CI −4.94 to −1.23; *p* = .001; Cohen's *d* = −0.46). Using the prespecified PHQ-9 cutoff for remission, 54% of participants in the intervention group achieved remission compared with 34% in the usual-care group (OR = 2.24, 95% CI 1.58–3.20). Although the between-group difference in PHQ-9 scores was below the approximately 5-point threshold commonly cited as a minimal clinically important difference for individual change, this threshold was developed primarily to interpret within-person change rather than between-group mean differences (57). The observed effect should therefore be considered alongside the moderate standardized effect size (d = −0.46) and the higher remission rate in the intervention group (56). Treatment adherence also improved more in the intervention group (AMD = 2.59, 95% CI 1.82–4.99; *p* = .035). At six months, mean adherence scores were 31.7 in the intervention group and 29.0 in the usual-care group, corresponding, on average, to high and moderate adherence, respectively, according to the scale's scoring criteria (56).

The trial provides preliminary evidence that trauma-informed principles can be integrated within collaborative care in routine primary care. However, its multicomponent design and comparison with usual care preclude determining the independent contribution of trauma-informed components or their incremental benefit over conventional collaborative care. The findings therefore inform the proposed TICC framework without validating it as a distinct treatment model.

### Implementation challenges and future directions

#### Implementation challenges

Integrating TICC into routine practice will require changes at clinical and organizational levels. Healthcare professionals will need competencies in assessing childhood maltreatment, trauma-informed communication, developmental case formulation, and relational approaches promoting safety, collaboration, and resilience. Sustaining these competencies will require interdisciplinary supervision and organizational support for staff well-being and reflective practice (34–36, 41–43). Implementation may be particularly challenging in primary care, where high demand, limited consultation time, workforce constraints, fragmented services, and competing priorities may hinder adoption and sustainability. These challenges may increase resource requirements and implementation complexity, making strong leadership, institutional commitment, and sustainable financing particularly important. Feasibility will vary across healthcare systems, especially in resource-constrained settings, and the incremental clinical benefit beyond well-implemented collaborative care has not yet been established (44, 45).

#### Future directions

Future research should determine whether TICC provides incremental clinical benefit beyond established collaborative care and which components, if any, contribute to improved outcomes. Direct comparative trials will therefore be particularly important. Research should also examine acceptability, adoption, fidelity, sustainability, resource requirements, and cost-effectiveness across healthcare systems and populations.

Another important question is whether the benefits of TICC vary across clinically meaningful patient groups. In particular, future studies should examine whether patients with extensive histories of childhood maltreatment or high perceived impact of childhood adversity derive greater benefit from trauma-informed adaptations, given their greater clinical complexity and poorer treatment outcomes observed in previous studies (9, 13). Such analyses should be prospectively specified whenever possible and formally evaluate treatment-effect heterogeneity rather than relying solely on within-subgroup comparisons.

#### Broader implications

The implications of TICC may extend beyond depression. Childhood maltreatment has been associated with multimorbidity, including chronic physical diseases, mental disorders, and increased healthcare utilization (58). Collaborative care has also benefited individuals with multimorbidity by improving mental health outcomes, quality of life, care coordination, and selected physical outcomes (59). Integrating trauma-informed principles may therefore help address complex biopsychosocial needs, but this possibility remains hypothetical and requires prospective evaluation across populations and settings.

### Conclusion

Collaborative care is one of the most extensively studied models for delivering evidence-based treatment for depression in primary care (8). However, the high prevalence of childhood maltreatment among individuals with depression, together with its association with greater clinical complexity and poorer treatment outcomes, suggests that some patients may have developmental and trauma-related needs that are not systematically addressed within conventional collaborative care (9, 13).

Rather than replacing collaborative care, TICC is proposed as an evidence-informed conceptual framework for strengthening this established model through the explicit integration of trauma-informed and developmental principles. It extends collaborative care by incorporating attention to childhood adversity when clinically appropriate, developmental case formulation, trauma-informed communication, and relational and organizational practices emphasizing safety, trustworthiness, collaboration, empowerment, and cultural responsiveness (11, 34, 35).

Preliminary evidence suggests that these principles can be integrated within a multicomponent collaborative care intervention and implemented in routine primary care (56). However, current evidence does not establish the independent contribution of trauma-informed components or their incremental clinical benefit beyond conventional collaborative care. TICC should therefore be regarded as a proposed conceptual framework rather than an empirically established treatment model. Future comparative trials should evaluate its effectiveness, cost-effectiveness, implementation requirements, and potential differential benefits across clinically meaningful patient groups. Such research will be necessary to determine whether trauma-informed adaptations can strengthen collaborative care and contribute to more personalized, developmentally informed, and contextually responsive depression care.

## Data Availability

No new data were generated or analyzed for this article. Further inquiries can be directed to the corresponding author.
